# Provisioning the origin and early evolution of life

**DOI:** 10.1042/ETLS20190011

**Published:** 2019-07-16

**Authors:** Long-Fei Wu, John D. Sutherland

**Affiliations:** Medical Research Council Laboratory of Molecular Biology, Francis Crick Avenue, Cambridge CB2 0QH, U.K.

**Keywords:** metabolism, origin of life, prebiotic chemistry

## Abstract

There is a lot of controversy in the origin and early evolution of life field, but most people agree that at the advent of genetically coded protein synthesis, cells must have had access to ribonucleotides, amino acids, lipids and some sort of energy source. However, the provenance of these materials is a contentious issue — did early life obtain its building blocks prefabricated from the environment, or did it synthesise them from feedstocks such as CO_2_ and N_2_? In the first case, synthesis conditions need not have been compatible with life and any kind of reaction network that furnished the building blocks — and not much else — could have provisioned the subsequent origin and early evolution of life. In the second case, synthesis must have been under life-compatible conditions, with the reaction network either along the same lines as extant biology or along different ones. On the basis of experimental evidence, we will argue in favour of prefabrication and against synthesis by life in its nascent state, especially synthesis that resembles extant biosynthesis, which we suggest would have been well-nigh impossible without biological catalysts.

## Introduction

Catalysis by enzymes and RNA is one of the wonders of biology. It is thought by many that RNA played a bigger role at the dawn of life than it does now, but once genetically coded proteins arrived on the scene, biology went into overdrive. So, what would have been required for early RNA replication and the emergence of translation? This difficult question becomes easier if we only think in material terms for there can be no doubt that ribonucleotides and amino acids, or derivatives thereof, were needed. Furthermore, lipids were most likely also needed as the natural selection required to optimise catalytic activities is most easily imaginable in the context of compartmentalisation by membranes. Four nucleotides are needed to endow RNA with a wide range of functions, but about half of the amino acids look late and functional proteins are possible with restricted ‘alphabets’ [1,2], so let us assume that around ten amino acids were also needed. Assuming membranes composed of a single lipid species, this means that about 15 simple to moderately complex compounds would have been necessary to progress biology towards translation. These chemical entities would have had to be abundant in the environment for primitive cells to feed off, or produced by cells in high yield — a modern bacterium primed for replication has remarkably high intracellular concentrations of amino acids (∼150 mM) and nucleotides (∼50 mM) [3]. There are those who suggest that impactors could have delivered these materials [4,5], but there are several compelling arguments against this. Big impactors, which have the potential to deliver significant amounts of material, vaporise on impact and their organic cargo is largely atomised, along with a portion of the surface and atmosphere of Earth, ultimately giving species such as NO^.^, CO and HCN [6,7]. Even in carbonaceous chondrites, which are the richest in ‘organics’, nucleotides have not been detected, levels of amino acids and lipids needed for life are very low and there are a huge number, likely millions, of other compounds present [8]. Such organic complexity effectively precludes subsequent chemical assembly of macromolecules destined to become biological because there is no means of selectively conjoining canonical components in the presence of myriad chemically similar, but non-canonical, components. So, efficient synthetic chemistry that could selectively make the canonical components from simple feedstocks on early Earth is our goal.

## What sort of chemistry?

We need to consider chemical feedstocks and conditions, and whether or not it is necessary to have any degree of physical separation of the various branches of the synthetic network, which makes the product tree, to enable all the products to be made. Biology's use of CHNOPS dictates the elemental composition of synthetic feedstocks needed. Water can provide some of the hydrogen and oxygen; hydrogen sulfide, the sulfur, and phosphate seems like a reasonable source of phosphorus — though solubility in the presence of certain metal ions is an issue — but carbon and nitrogen could come from quite different sources. In extant biology, the average oxidation level of carbon is around that of the carbon in formaldehyde, but nitrogen is for the most part fully reduced. Separate fixation of these elements from CO_2_ and N_2_, the way biology now does it, thus requires a lot of reduction, but simultaneous fixation of both elements from HCN would require less reduction. Some shy away from hydrogen cyanide as a feedstock on the grounds of its toxicity [9], but the target of cyanide, cytochrome C oxidase — responsible for electron transport to oxygen at the end of the respiratory chain — post-dates the oxygenation of the atmosphere by highly evolved life starting roughly two and a half billion years ago, at least a billion years after the origin of life. Furthermore, with respiratory chain alterations, certain microbial organisms can not only tolerate cyanide, but thrive on it as a nitrogen and carbon source [10–12].

**Figure 1.**
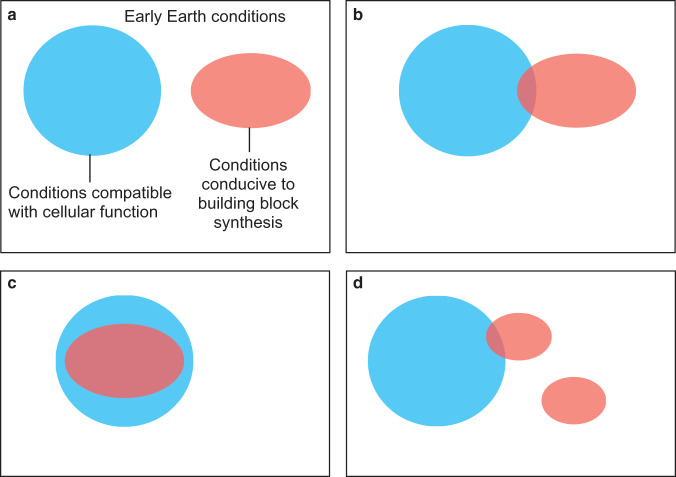
To what extent do life-compatible conditions overlap conditions for the synthesis of (proto)biological building blocks? Four representative possibilities are shown: (a) no overlap, (b) limited overlap, (c) complete overlap, and (d) two different sets of conditions are needed for building block synthesis, one set partly overlaps life-compatible conditions and the other not at all.

What about the conditions under which such feedstocks could react together to produce our 15 or so simple to moderately complex building blocks? Early Earth offers a range of environments with either changing or relatively constant conditions. Precipitation and evaporation enable wet–dry cycles, geothermal and impact heating allow high temperatures to be attained, low solar luminosity allows a snowball Earth, solar UV irradiation reaches the surface — though wavelengths below ∼200 nm are attenuated by CO_2_ and water vapour in the atmosphere — and high pressures are possible sub-surface and in the depths of the oceans. Thus, without any constraint, there are numerous conditions and sequences of conditions possible on early Earth. However, if synthesis had to be compatible with life, then certain conditions are effectively ruled out, for example, heating above the boiling point of water, UV irradiation and so on. Yes, there are plenty of extremophiles that can cope with all sorts of hostile environments on Earth now [13], but in their cellular interiors water is liquid and penetrating UV irradiation is lethal (above a certain level). Given all of this, we now need to consider the extent to which synthesis conditions might have matched those consistent with emerging life (Figure 1). *A priori*, it is possible that synthesis conditions were completely incompatible with life, or partially, or fully, compatible. If different conditions were needed for different branches of the synthetic network, either set could have had the same degree of overlap with the conditions pertaining in nascent biological systems. One might have a gut feeling one way or the other, but surely it is worth amassing a large body of evidence from synthetic chemical investigations, so an assessment can be made *a posteriori*? Throwing such caution aside, however, and without comprehensive experimentation, some people have made their minds up that the synthetic network that furnishes the building blocks must ‘resemble extant biochemistry in terms of substrates, reaction pathways, catalysts and energy coupling’ [14]. Proclaiming that because biology does it this way now, it must always have done it this way, is akin to a person living in a future completely powered by renewable resources assuming that previous generations must always have lived that way. It is a denial of the possibility of adaptation. In the same way that society is beginning to wean itself off fossil fuels, biology could have gradually adapted from consuming dwindling environmental supplies of amino acids, nucleotides and lipids to making its own, *in cellulo*, from renewable resources. *Adaptation is a defining feature of life, the hydrogenation of CO_2_ is not*.

To cut a long story short, organic chemists have pieced together reaction networks that make about half of the canonical amino acids, the pyrimidine and purine ribonucleotides and diacyl-glycerol-phosphate lipids [15–24]. On the basis of what is known to date, these chemists have made three conclusions that are germane to our argument:

(i)  Hydrogen cyanide is a perfect feedstock to produce the palette of products necessary for the emergence of translation. It is a source of carbon and nitrogen and is constitutionally implicated in the purines (adenine is a pentamer of HCN), the amino acids (through Strecker-type syntheses), sugars and glycerol (through reductive homologation).
(ii)  Certain reactions work best under anhydrous conditions, for example, nucleoside and glycerol phosphorylation, and glycosidation. Other reactions require UV irradiation or heating above the boiling point of water.
(iii) It is not possible to make everything in ‘one pot’ by one sequence of conditions, some degree of separation of the branches of each reaction network is necessary.

**Figure 2.**
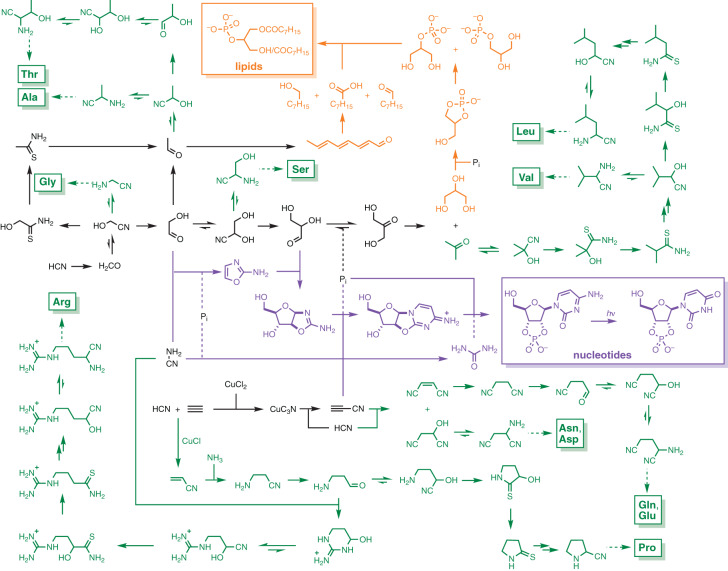
Cyanosulfidic protometabolic reaction network leading from HCN and derivatives thereof to pyrimidine ribonucleotides, and amino acid and lipid precursors. Pathways, intermediates and products specific to a particular product class are shown in colour (pyrimidine ribonucleotides, purple; amino acids, green; lipids, orange) with products boxed, shared starting materials, pathways and intermediates are shown in black.

In our contribution to this (Figure 2) [18–21,24], the pivotal importance of HCN is especially evident — every carbon and nitrogen atom of the final set of products can ultimately derive from HCN. The set of products is directed by the inherent reactivity of HCN and intermediates derived therefrom under a geochemically plausible sequence of conditions. Environmental energy in the form of heat and light supplements the chemical energy inherent in the triple bond of HCN to drive the reactions of our network. Prefabrication is thus comprehensively supported by experimentation. But it is not accepted by everyone, because it ‘does not narrow the gap between prebiotic chemistry and biochemistry’ [14]. Aside from the fact that the gap does not need narrowing because adaptation can traverse it, there are additional reasons to refute alternatives to prefabrication. We need to examine what would be required for early synthesis to ‘resemble extant biochemistry in terms of substrates, reaction pathways, catalysts and energy coupling’ [14].

**Figure 3.**
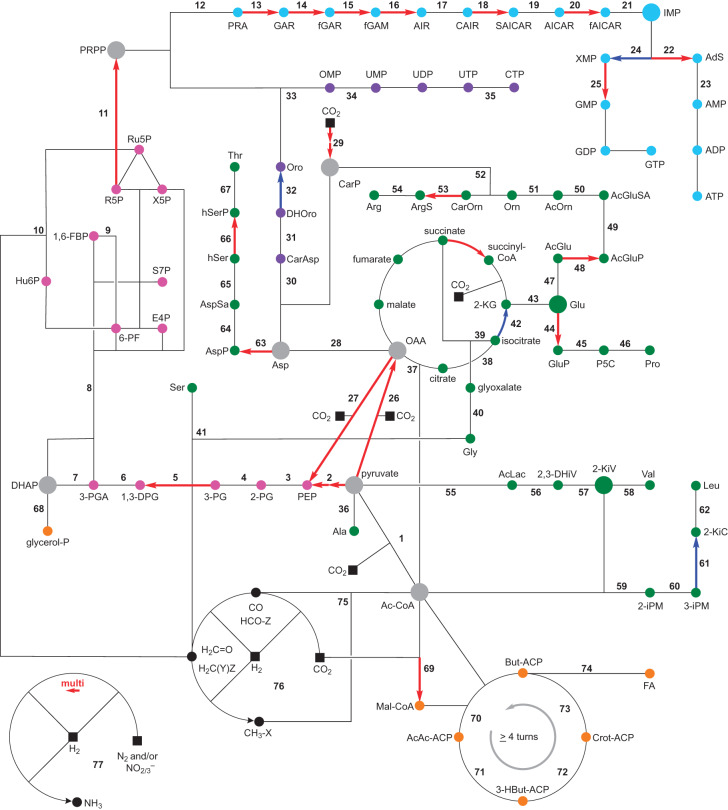
Minimal biosynthetic reaction network leading from CO_2_ and N_2_ (or 
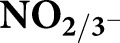
) to ribonucleotides, amino acids and lipid precursors. Hub compounds are indicated by large disks and other compounds are indicated by smaller disks coloured according to biosynthetic route/product class (partial gluconeogenesis, pink; purine ribonucleotides, light blue; pyrimidine ribonucleotides, purple; amino acids, green; lipids, orange). Feedstocks are indicated by black squares and the immediate metabolites thereof as small black disks. ATP(/GTP)-consuming reactions are shown as red arrows, the number of which matches the number of high-energy phosphate bonds consumed per reaction. Oxidation reactions are shown by blue arrows. A more complete description of each reaction is provided in the Supplementary Material, as is a list of abbreviations.

According to this mantra, a reaction network — starting from CO_2_ and N_2_, or other inorganic nitrogen sources — of the sort shown in Figure 3 would be needed to make a similar set of products to that which we and others have made from HCN [25]. Extant biology effects these ∼75 different reactions, and many others, using enzymes of extraordinarily high catalytic prowess and specificity in ‘one pot’ — the cell — under common conditions. Essentially, CO_2_ is fixed by reduction to CO and a methyl group attached to a cofactor and these two entities are then coupled together to give, after thiolysis, acetyl-CoA. Reductive coupling of acetyl-CoA to another CO_2_ then gives pyruvate, which is a major branch point. One branch therefrom leads through partial gluconeogenesis to phosphoribosyl pyrophosphate (PRPP) a key ribonucleotide precursor, another feeds into the (reductive) citric acid cycle, a third leads to leucine and valine and a fourth to alanine. Oxaloacetate, the entry point of the citric acid cycle, is also an exit point, being converted to aspartate by transamination. This amino acid is not just one of the key products, but also another branch point, precursing both the nucleobase moiety of the pyrimidine ribonucleotides and threonine. The citric acid cycle also furnishes the amino acids glycine and thence serine, glutamate, proline and arginine. Carbamoyl phosphate, formed from CO_2_ and ammonia, provides the fully oxidised carbon of the pyrimidine ribonucleotides and arginine. Finally, the lipid constituents, glycerol-3-phosphate and fatty acids, are formed from the triose phosphates and acetyl-CoA, respectively. Every single reaction is associated with sophisticated and beautiful enzymology, the co-ordinated operation of which is quite simply breathtaking. But, there are people who insist that the whole network could operate without enzymes sufficiently well to provide for the needs of emerging life [14,26,27]. Should we believe them? In short, no, because there are eight fundamental and irredeemable problems associated with the operation of this network without enzymes:

(i)   Catalysis, catalysis, catalysis. For very few enzymes is the underlying reaction rate significant — if it were, regulation would be impossible as the reaction could not be switched off as necessary. The degree of catalysis by enzymes can be remarkable, for example, orotidine monophosphate decarboxylase accelerates the uncatalysed reaction, which has a half-life of seventy eight million years, ∼10^17^-fold [28]. Without this single reaction, there would be no pyrimidine ribonucleotides produced by the (proto)biological network, no functional RNA and no prospects of translation ever emerging. Catalysis of almost all of the reactions of the network would thus be necessary for the network to operate with any degree of efficiency. For some enzymes that use cofactors (orotidine monophosphate decarboxylase does not), limited catalysis is observed for the cofactor alone [29], but the crucial organic (redox) cofactors such as nicotinamide and flavin derivatives, thiamin pyrophosphate (TPP), biotin and the corrinoids would not be present — unless they too were synthesised by the system, in which case add scores of additional, difficult reactions to the network. Thiamin pyrophosphate in particular is crucial and enzymes that deploy it as an umpolung catalyst can bring reactions with billion year uncatalysed half-lives into play in biology [28].
(ii) Negative aspects of catalysis. Metal ion cofactors catalyse deleterious as well as desired reactions. It has been known for decades that metal ions, particularly divalent ones, can catalyse certain biological reactions, albeit modestly [30], but without shepherding by enzymes they also catalyse unwanted reactions. An essential reaction of the partial gluconeogenesis branch is the enolase-catalysed conjugate addition of water to phosphoenolpyruvate (PEP), giving 2-phosphoglycerate (2-PG), but divalent metal ions instead catalyse the attack of water at the phosphate group of PEP resulting in hydrolysis back to pyruvate [31]. Certain ions are particularly effective at this, the hydrolysis being ‘strongly accelerated’ by ferrous ions [26], for example. Likewise, the decarboxylation of oxaloacetate back to pyruvate is catalysed by divalent metal ions [32], as is the aldol dimerisation of pyruvate [33] which would compete with its conversion to acetolactate — even if that could ever be achieved without TPP — and thence valine and leucine.
(iii)  Flux control from branch points and regulation. Flux through the different branches emanating from branch points has to be controlled such that all the branches are productive. Thus, if any of the reactions leading away from pyruvate is significantly faster than the others, the others suffer, yet all are crucial. This problem is compounded by the divalent metal ion problem outlined above. Biology catalyses reactions differentially such that most are brought up to a similar speed [28], thus avoiding bottlenecks, but the background reactions have widely differing rates. Thus, even if prebiotically plausible catalysts for all 75 or so reactions could be found, it would not be enough, as they would additionally need to accelerate the individual reactions in the same differential way as do the enzymes used in biology, for the network to operate with the efficiency needed.
(iv) Instability of intermediates. Evolution has resulted in biology being able to handle some extremely unstable intermediates, which decompose in the absence of enzymes. Thus, for example, phosphoribosylamine, produced by ammonolysis of PRPP, hydrolyses to ribose-5-phosphate with a half-life of 38 seconds under physiological conditions and it anomerises even faster [34]. Any non-enzymatic catalyst for the subsequent glycylation step would have to outcompete these rapid reactions or there would be no purine ribonucleotides.

Enolisation is required for the interconversion of the triose phosphates, but the elimination of phosphate from the enolate of glyceraldehyde-3-phosphate is facile, thus the triose-3-phosphates are intrinsically unstable [35]. Nature gets round this by having triose phosphate isomerase both positively catalysing enolisation and negatively catalysing the elimination. In thermophiles, the uncatalysed elimination becomes problematic with the triose phosphates only having a half-life of minutes at 80°C [36]. These microorganisms therefore have a bifunctional fructose-1,6-bisphosphate aldolase-fructose-1,6-bisphosphatase to overcome this and trap them as the more stable fructose-6-phosphate. A non-enzymatic catalyst for this combination of aldolisation and phosphate ester hydrolysis seems an extraordinarily unrealistic prospect

(v)    Oxidation in a reducing environment. Biology plays redox chemistry really well, but achieving this at a network level without enzymes and redox cofactors would be extremely tricky. For sure, oxidation and reduction reactions can operate simultaneously in chemistry, but oxidising the hydroxyl group of 3-isopropylmalate, *en route* to leucine, would be challenging in the presence of other alcohols, particularly whilst simultaneously reducing dihydroxyacetone phosphate to glycerol-3-phosphate. Dehydrogenating dihydroorotate and inosine monophosphate during ribonucleotide synthesis would be similarly difficult whilst hydrogenating enoyl derivatives in fatty acid synthesis.
(vi)    Substrate selectivity. By employing enzymes with concave active sites, biology is capable of discriminating between chemically similar compounds of different shapes and sizes. Without such discrimination, it would not be possible to control selectivity in the network. Thus, for example, any non-enzymatic phosphatase mimic that could hydrolyse fructose-1,6-bisphosphate, and mitigate the triose phosphate instability problem alluded to above, would be hard-pressed not to dephosphorylate any of the many monophosphates in the network, especially dihydroxyacetone phosphate. Similarly, in purine ribonucleotide synthesis any simple catalyst that happened to be able to formylate the amino group of aminoimidazolecarboxamide ribonucleotide (AICAR) would have a hard job in not formylating the more nucleophilic amino group of the closely related aminoimidazole ribonucleotide (AIR). Formylation of the latter intermediate would block purine ribonucleotide synthesis and thus prevent the formation of functional RNA.
(vii)   The ammonia problem. Ammonia is needed throughout the network, but there are many intermediates which are destroyed by this potent nucleophile. Biology gets round this by storing ammonia in compounds such as glutamate and glutamine and then releasing it at the enzyme active sites [37], or at the entrances to tunnels to other active sites [38], where it is needed. Absent this trick, it would be highly difficult to reductively aminate pyruvate to alanine, for example, without ammonolysing the various thioesters and acyl phosphates of the network. Ammonolysis of acetyl-CoA to give acetamide would shut down the synthesis of pyruvate. Ammonolysis of acyl phosphates, or thioesters derived therefrom, would prevent the reduction of carboxylate groups to aldehydes that occurs throughout the network.
(viii)  Energy coupling. Expenditure of ATP (or GTP) is required to drive many of the reactions of the network, which would otherwise be energetically unfavourable. Proponents of building block synthesis by nascent life have gradually moved away from the idea that some sort of primitive chemiosmosis could regenerate ATP (and thence GTP). They have even moved away from nucleoside triphosphates as an energy currency because of their kinetic inertness and currently seem to favour acetyl phosphate as a source of energy [14,39], even though its hydrolysis is catalysed by metal ions [40]. Granted, acetyl phosphate has the potential to phosphorylate homoserine, for example, — though it would have to be a very selective catalyst that managed to discriminate between the hydroxyl group of this alcohol and 55 M water — but what about the crucial phosphorylation of pyruvate to PEP? This latter reaction needs two high-energy phosphate bonds of ATP to be spent to drive it and the enzyme that catalyses the transformation does so using some pretty spectacular enzymology [41]. It is an almost inconceivable transformation using acetyl phosphate and prebiotically plausible non-enzymatic catalysts. Even if it was possible, it would have to compete with the divalent metal ion-catalysed hydrolysis back to pyruvate [32].

Are there any ways to escape from these eight fundamental problems? If the mantra is strictly adhered to, no. So, what is now happening is that people who insist on synthesis *in cellulo* as biology emerges are opting for reactions that are increasingly different from those in extant biology. But the eight problems are irredeemable even by this shifting of the goal posts. Thus, one cannot invoke iron-nickel sulfide catalysed synthesis of acetyl thioesters [42] at the same time and place as the ferrous–ferric iron-mediated reductive amination of pyruvate to alanine with 0.375 M ammonia [43] because of the ammonia problem. It is not possible to have a formose reaction of formaldehyde to generate pentoses [44] at the same time as ferrous iron-mediated aldolisation chemistry of pyruvate [27] because of myriad deleterious crossed aldolisations between oxoacids, formaldehyde and sugars. One cannot rely on metallic iron and hydroxylamine as a way of converting pyruvate to alanine [27] because the same conditions will destroy key intermediates in the network, for example by converting all acyl phosphates and thioesters to hydroxamates. No amount of hard selling can resurrect this dead parrot of an idea — *caveat emptor*.

Which brings us back to the idea that HCN chemistry made the 15 key products which enabled the origin and earliest evolution of life. An energy source would also have been necessary to drive energy-dissipative macromolecular assembly cycles from the monomers and maintain the system in an out of equilibrium state. Simple isonitriles are being investigated in this regard [45], they are also easily produced from HCN and can activate both phosphate and carboxylate groups as would have been essential. Provisioned and powered by the HCN product tree, early biology could have ‘lived off the fat of the land’. But extant biology, does not live this way. So how could the transition to the biosynthetic network with catalysed energy coupling take place? Considering the material aspect of this problem first, as biology spread, resources would have become depleted and there would have been a need to start to make the key 15 products within cells as part of biochemistry. We cannot know for sure which were the first biosynthetic reactions, but it is easy to imagine that ribonucleotides became scarce in the environment early on. However, as a by-product of prebiotic ribonucleotide syntheses involving photochemistry [20,21], for example, the environment would have likely contained nucleobases and maybe even sugar phosphates. This immediately hints at the salvage pathways of ribonucleotide biosynthesis wherein the canonical nucleobases are ribosylated. This cuts out many steps of the *de novo* biosyntheses, in particular, the long, difficult and very energetically demanding sequence of steps from PRPP to the purine ribonucleotides. That nascent biology battled its way through this arduous sequence before it benefitted from ‘homemade’ purine ribonucleotides supplementing diminishing environmental supplies, simply beggars belief. It seems much more likely that a prefabricated purine was bolted on to a homemade sugar phosphate, or that both components were prefabricated and then put together IKEA-fashion in early cells. So, could a primitive ribozyme or ribonucleoprotein catalyse nucleobase ribosylation? The answer is not just a resounding yes, ribozymes can do it [46], but also that they are ideally suited to do it by a dissociative mechanism because they can use the negative charge of their backbone to stabilise developing positive charge in the nucleosidation reaction [47]. However, the ribozyme catalyst cannot stop some competing hydrolysis because ribozymes cannot envelop their substrates the way proteins can. So, there would have been an opportunity for a ribonucleoprotein based on our restricted amino acid alphabet to do even better and pull biology back from the brink. Other reactions could have been added to build up the biosynthetic network in a patchwork fashion [48] by virtue of the likely catalytic promiscuity of early (ribonucleo)proteins. This is the stage at which the otherwise inefficient and non-specific metal ion-catalysed reactions are brought into play with enzyme evolution restricting specificity and improving catalysis. What about energy coupling? Nucleoside triphosphates could have accumulated as by-products of nucleoside monophosphate activation then chance catalysis of ATP usage to drive a thermodynamically uphill reaction would have taken the system to another level. Depletion of ATP stocks could then have led to substrate level phosphorylation. Adaptation to the way biology now does it would have begun—the rest is history.

## Summary

- Synthesising life's building blocks using the same pathways and intermediates as extant biology is a near impossible task without enzymes.
- However, the building blocks can be synthesised from hydrogen cyanide and its derivatives under prebiotically plausible conditions, but some of these conditions are incompatible with life.
- Therefore, it is proposed that the origin and early evolution of life took place in an environment containing previously synthesised building blocks.
- The pathways and intermediates of extant biology could then have been introduced in a patchwork fashion as environmental supplies of building blocks became depleted and early organisms adapted to their changing conditions.

## Abbreviations

2-PG: 2-phosphoglycerate
AICAR: aminoimidazolecarboxamide ribonucleotide
AIR: aminoimidazole ribonucleotide
PEP: phosphoenolpyruvate
PRPP: phosphoribosyl pyrophosphate
TPP: thiamin pyrophosphate
