# Supplementary material for: Provisioning the origin and early evolution of life
Source: Emerg Top Life Sci. 2019 Jul 16;3(5):459–68. doi: 10.1042/ETLS20190011 (PMC6992421; doi:10.1042/ETLS20190011)
Supplement: Supplementary Material [file ETLS-3-459-s1.pdf]

Supplementary Material.

Abbreviations

Abbreviation	Full Name	Reaction number
1,3-DPG	1,3-Diphosphoglycerate	5
1,6-FBP	Fructose-1,6-bisphosphate	8
2-iPM	2-Isopropylmalate	59
2-KG	2-Ketoglutarate	42
2-KiC	2-Ketoisocaproate	61
2-KiV	2-Ketoisovalerate	57
2-PG	2-Phosphoglycerate	3
2,3-DHiV	2,3-Dihydroxyisovalerate	56
3-HBut-ACP	3-Hydroxylbutanoyl-acyl carrier protein	71
3-iPM	3-Isopropylmalate	60
3-PG	3-Phosphoglycerate	4
3-PGA	Glyceraldehyde-3-phosphate	6
6-PF	Fructose-6-phosphate	9
AcAc-ACP	Acetoacetyl-acyl carrier protein	70
AcCoA	Acetyl coenzyme A	75
AcGlu	N-Acetylglutamate	47
AcGluP	N-Acetyl-L-glutamyl-5-phosphate	48
AcGluSa	N-Acetyl-L-glutamate-5-semialdehyde	49
AcLac	2-Acetolactate	55
AcOrn	N^2-Acetylornithine	50
AdS	Adenylosuccinate	22
AICAR	Aminoimidazole carboxamide ribonucleotide	19
AIR	5'-Phosphoribosyl-5-aminoimidazole	16
Ala	L-Alanine	36
AMP	Adenosine-5'-monophosphate	23
Arg	L-Arginine	54
ArgS	L-Argininosuccinate	53
Asp	L-Aspartate	28
AspP	L-Aspartyl-4-phosphate	63
AspSa	L-Aspartate-4-semialdehyde	64
But-ACP	Butyryl-acyl carrier protein	73
CAIR	5'-Phosphoribosyl-4-carboxy-5-aminoimidazole	17
CarASP	N-Carbamyl-L-aspartate	30
CarOrn	Citrulline	52
CarP	Carbamoyl phosphate	29
Crot-ACP	Crotonyl-acyl carrier protein	72

CTP	Cytidine-5'-triphosphate	35
DHAP	Dihydroxyacetone phosphate	7
DHOro	Dihydroorotate	31
E4P	Erythrose-4-phosphate	
FA	Fatty acid	74
fAICAR	5-Formamidoimidazole-4-carboxamide ribonucleotide	20
fGAM	Formylglycinamidine-ribonucleotide	15
fGAR	N^2-Formyl-N^1-(5-phospho-D-ribosyl)glycinamide	14
GAR	N^1-(5-phospho-D-ribosyl)glycinamide	13
Glu	L-Glutamate	43
GluP	L-Glutamyl-5-phosphate	44
Gly	Glycine	40
Glycerol-P	Glycerol-3-phosphate	68
GMP	Guanosine-5'-monophosphate	25
hSer	L-Homoserine	65
hSerP	O-Phospho-L-homoserine	66
Hu6P	3-Hexulose-6-phosphate	10
IMP	Inosine-5'-monophosphate	21
Leu	L-Leucine	62
Mal-CoA	Malonyl-CoA	69
OAA	Oxaloacetate	26, 27
OMP	Orotidine-5'-monophosphate	33
Orn	Ornithine	51
Oro	Orotate	32
P5C	Δ^1-Pyrroline-5-carboxylate	45
PEP	Phosphoenolpyruvate	2
PRA	Phosphoribosylamine	12
Pro	L-Proline	46
PRPP	Phosphoribosylpyrophosphate	11
R5P	Ribose-5-phosphate	
Ru5P	Ribulose-5-phosphate	
SAICAR	Succinyl-aminoimidazolecarboxamide ribonucleotide	18
S7P	Sedoheptulose-7-phosphate	
Ser	L-Serine	41
Thr	L-Threonine	67
UMP	Uridine-5'-monophosphate	34
Val	L-Valine	58
X5P	Xylulose-5-phosphate	
XMP	Xanthosine-5'-monophosphate	24

More detailed description of the reactions comprising the network Figure 3

1. Pyruvate ferredoxin oxidoreductase. A multistep reaction in which the acetyl group of acetyl-CoA is transferred to thiamine pyrophosphate (TPP) and reduced by ferredoxin to

give hydroxyethyl-TPP which is then carboxylated to a new adduct which equilibrates with pyruvate and TPP:

Acetyl-CoA + TPP + $2H^+$ + Fd_{red}^{2-} = He-TPP + CoASH + Fd_{ox}
He-TPP + CO_2 = pyruvate + TPP + H^+

The electron transfer from the reduced ferredoxin is not direct but is via enzyme-bound iron-sulfur clusters. This highly complex reaction requiring umpolung is difficult to imagine without cofactors and the enzyme under reasonable conditions.

2. Phosphoenolpyruvate synthetase. Two high-energy phosphate bonds need to be spent to drive the conversion of pyruvate to phosphoenolpyruvate (PEP). This enzyme achieves this by way of both pyrophosphoryl- and phosphoryl-enzyme intermediates using some pretty spectacular enzymology [4]:

ATP + enzyme = AMP + enzyme-$P_\beta P_\gamma$
H_2O + enzyme-$P_\beta P_\gamma$ = $P_{\gamma i}$ + enzyme-P_β
pyruvate + enzyme-P_β = PEP + enzyme

One equivalent of acetyl phosphate could not drive this conversion thermodynamically and hoping to achieve it with two equivalents without an enzyme is a pipe dream. A potential alternative would go by way of oxaloacetate and reactions 26 and 27, but achieving this non-enzymatically is fraught with difficulties, *vide infra*.

3. Enolase. Addition of the poorly nucleophilic hydroxide ion to the alkene of PEP generating an unstable carbanion which then undergoes protonation giving 2-phosphoglycerate:

PEP + H_2O = 2-PG

PEP undergoes non-enzymatic hydrolysis rather than hydration. The hydrolysis reaction is catalysed by metal ions [31], in particular being 'strongly accelerated' by ferrous ions [26].

4. Phosphoglycerate mutase. The phosphate is shuttled between the 2- and 3-positions of glycerate via a phosphoryl-enzyme intermediate:

2-PG + enzyme = glycerate + enzyme-P
glycerate + enzyme-P = 3-PG + enzyme

Without an enzyme, the removal of phosphate in the first step would equate to hydrolysis to glycerate and inorganic phosphate.

5. Phosphoglycerate kinase. Substrate-selective carboxylate phosphorylation:

3-PG + ATP = 1,3-DPG + ADP

Free 1,3-diphosphoglycerate is very unstable having a half-life for non-enzymatic hydrolysis to 3-phosphoglycerate of about 30 mins at room temperature and so it is reversibly passed to the next enzyme via an enzyme-substrate-enzyme complex [S1].

6. Glyceraldehyde-3-phosphate dehydrogenase. Covalently bound thioester intermediate and redox by nicotinamide mediated hydride transfer giving a hemithioacetal of glyceraldehyde-3-phosphate which equilibrates with the free aldehyde:

1,3-DPG + HS-enzyme = P_i + 3-PG-S-enzyme
3-PG-S-enzyme + NAD(P)H + H^+ = 3-PGA + HS-enzyme + $NAD(P)^+$

There is an enzyme that catalyses the direct oxidation of glyceraldehyde-3-phosphate to 3-phosphoglycerate, but this reaction is, to all extents and purposes, irreversible and so a non-enzymatic variant could not operate in the gluconeogenetic direction.

7. Triosephosphate isomerase. Positive catalysis of enolisation coupled to negative catalysis of phosphate elimination:

3-PGA = DHAP

Attempted isomerisation of the triose phosphates in the absence of enzymes invariably results in elimination of phosphate by an $E1_{cB}$-irreversible mechanism and generation of methylglyoxal [35]. This elimination is extremely rapid at high temperatures [36].

8. Fructose-1,6-bisphosphate aldolase. Selective crossed aldol reaction:

DHAP + 3-PGA = 1,6-FBP

In the absence of enzyme catalysis, background aldol reaction would compete with a myriad other aldol reactions, particularly enolate hydroxymethylation in the presence of formaldehyde.

9. Fructose-1,6-bisphosphatase: Highly selective phosphatase activity that offsets the otherwise unfavourable thermodynamics of partial gluconeogenesis:

1,6-FBP + H_2O = 6-PF + P_i

Without selective catalysis, a general background phosphatase activity would be energetically disastrous as it would deplete all phosphate monoesters. A bifunctional enzyme catalysing this reaction and the preceding one is commonly found in thermophilic autotrophic microorganisms, 'its bifunctionality ensures that heat-labile triosephosphates are quickly removed and trapped in stable fructose-6-phosphate'. The half-life of triosephosphates at 80 ^oC, pH 7, was confirmed to be 4 min. [36].

10. 3-Hexulose-6-phosphate synthase. Regioselective hydroxymethylation of ribulose-5-phosphate:

Ru5P + CH_2O = Hu6P

Given the large number of background enolisation reactions invoked, hoping for hydroxymethylation of just one enediolate is an appeal to magic.

11. Phosphoribosylpyrophosphate synthetase. Regioselective pyrophosphorylation of one anomer of the furanose form of ribose-5-phosphate:

R5P + ATP = PRPP + AMP

Another reaction that involves attack at the β-phosphate of ATP. Achieving the same conversion with acetylphosphate would require initial phosphorylation of one anomer of the furanose form of ribose-5-phosphate followed by regioselective phosphorylation of one of the phosphate groups of 5-phosphoribosylphosphate. This second phosphorylation would also have to be substrate-selective to avoid the phosphorylation of pretty much all other phosphate monoesters. PRPP is chemically unstable, particularly in the presence of divalent metal cations and decomposes to ribose-5-phosphate, 5-phosphoribosyl 1,2-cyclic phosphate and ribose-1,5-cyclic phosphate [S2].

12. Glutamine phosphoribosylpyrophosphate amidotransferase. Ammonolysis of PRPP to phosphoribosylamine using ammonia generated *in situ* by hydrolysis of glutamine to glutamate:

PRPP + Gln + H_2O = PRA + Glu + PP_i

Under physiological conditions, PRA hydrolyses to ribose-5-phosphate with a half-life of 38 s and it anomerises even faster [34].

13. Glycinamide ribonucleotide synthetase. Reaction of glycine with ATP to give glycyl phosphate followed by glycylation of the β-anomer of PRA giving glycinamide ribonucleotide:

Gly + ATP = GlyP + ADP
GlyP + PRA = GAR + P_i

To overcome hydrolysis of PRA it is thought that it is channelled to this enzyme from the previous enzyme in the pathway [S3]. Acetyl phosphate is not sufficiently high in energy to phosphorylate glycine to a significant extent. Another transformation that would be near impossible in the absence of an enzyme.

14. Glycinamide ribonucleotide transformylase. Formylation of GAR using N10-formyltetrahydrofolate or formate and ATP - arguably the latter is simpler:

f + ATP = fP + ADP
fP + GAR = fGAR + P_i

Formylphosphate is unstable undergoing hydrolysis by nucleophilic attack at both carbon and phosphorus. Synthesis of formylphosphate from formate and acetyl phosphate would be unfavourable because of the lower pK_a of formic acid compared to acetic acid.

15. Formylglycinamidine ribonucleotide synthetase. Amidination of the glycinamide amide using ammonia generated in situ by hydrolysis of glutamine to glutamate and using ATP to phosphorylate the amide oxygen such that it is converted into a better leaving group than PRA:

fGAR + Gln + H_2O + ATP = fGAM + Glu + ADP + Pi

Absent an enzyme, the glycinamide amide of fGAR is less reactive than the formyl amide. Without the phosphorylation, ammonolysis would first deformylate fGAR to GAR then cleave GAR to PRA which would hydrolyse to ribose-5-phosphate.

16. Aminoimidazole ribonucleotide synthetase. Attack of N1 of fGAM at the formyl carbon with phosphorylation of the formyl oxygen to render it a better leaving group than nitrogen:

fGAM + ATP = AIR + ADP + P_i

Even if the formamido group could be activated in water without an enzyme – a remote possibility – cyclisation by attack of the unsubstituted amidine nitrogen rather than N1 would be expected.

17. Aminoimidazole ribonucleotide carboxylase: Reversible formation of the carbamate by N-carboxylation of aminoimidazole ribonucleotide followed by enamine C-carboxylation:

AIR + CO_2 = CAIR

At low pCO_2, ATP is used to drive the initial N-carboxylation becoming ADP and P_i in the process. One of the only reactions in this whole scheme to proceed at an appreciable rate without deleterious side reactions in the absence of catalysis although only at high pCO_2 when the enzyme catalysed conversion is still a million times faster [S4]!

18. Succinyl-aminoimidazolecarboxamide ribonucleotide synthetase. Phosphorylation of CAIR to the acyl phosphate which is then used to acylate the amino group of aspartate:

CAIR + ATP = CAIRP + ADP
CAIRP + Asp = SAICAR + P_i

Without a substrate-selective catalyst, acylation of other amino acid amino groups eg. glycine would be inevitable, this would not allow the subsequent elimination that results in overall amidation.

19. Adenylosuccinate lyase activity I. Elimination of a poor leaving group from SAICAR by concerted general acid-base catalysis by two precisely positioned histidine side chains:

SAICAR = AICAR + fumarate

20. Aminoimidazolecarboxamide ribonucleotide transformylase. Thermodynamically uphill formylation of the poorly nucleophilic amino group of AICAR using N^{10}-formyltetrahydrofolate.

$AICAR + fFH_4 = fAICAR + FH_4$

Formylation of AIR, which has a much more nucleophilic amino group, would prevent N- and C- carboxylation and thereby prevent purine ribonucleotide synthesis.

21. Inosine monophosphate cyclohydrolase. Closure of the purine 6-membered ring by attack of the carboxamide nitrogen on the formyl group, possibly assisted by orbital steering, followed by proton transfers and elimination of water:

$fAICAR = IMP + H_2O$

22. Adenylosuccinate synthetase. Phosphorylation of the 6-oxo group of IMP followed by attack of the imidoyl phosphate on carbon by the amino group of aspartate and displacement of inorganic phosphate:

IMP + GTP = PIMP + GDP
PIMP + Asp = AdS + P_i

Reaction of the amino groups of other amino acids eg. glycine, would be unpreventable in the absence of an enzyme. These inevitable side products could not undergo the subsequent elimination that results in overall amination.

23. Adenylosuccinate lyase activity II. Elimination of a poor leaving group from AdS by concerted general acid-base catalysis by two precisely positioned histidine side chains:

AdS= AMP + fumarate

24. Inosine monophosphate dehydrogenase. Regiospecific oxidation of C2 of IMP by NAD^+ with concomitant addition of water:

$IMP + NAD^+ + H_2O = XMP + NADH + H^+$

A difficult reaction in a reducing environment. Without an enzyme to control the specificity, other oxidants could oxidise the diol or imidazole ring.

25. Guanosine monophosphate synthetase. Reversible adenylation of O2 of XMP followed by aminolysis by attack of the amido nitrogen of glutamine and carboxamide hydrolysis, or less favourably by free ammonia - the latter is less complex:

XMP + ATP = AdXMP + PP_i
$AdXMP + NH_3 = GMP + AMP$

Another incredibly challenging transformation in water without an enzyme – O2 of XMP would have to outcompete 55 M water to react with an activating agent just to accomplish the first step.

26. Pyruvate carboxylase. This enzyme uses ATP to couple bicarbonate and biotin-carboxyl carrier protein to give N^1-carboxybiotin-CCP which then carboxylates the enolate of pyruvate to give oxaloacetate:

Biotin-CCP + HCO_3^- + ATP = ^-O_2C-biotin-CCP + ADP + P_i
pyruvate + ^-O_2C-biotin-CCP = OAA + biotin-CCP

Another evolutionary triumph whereby ATP hydrolysis is used to drive an extremely thermodynamically uphill reaction – any primitive version of this reaction would have to battle the spontaneous decarboxylation of oxaloacetate which would lead to an energy draining futile cycle. The spontaneous decarboxylation is catalysed by divalent metal ions [32].

27. Phosphoenolpyruvate carboxykinase. Decarboxylation of oxaloacetate gives the enolate of pyruvate which is phosphorylated by GTP:

OAA + GTP = PEP + GDP + CO_2

Competing protonation of the enolate by solvent is prevented by a water-tight enzyme active site – how could this possibly be achieved in water without an enzyme?

28. Transaminase or amino acid dehydrogenase. Transamination from glutamate shifts the ammonia assimilation problem so the dehydrogenase is potentially simpler:

OAA + NH_3 + NAD(P)H + H^+ = Asp + $NAD(P)^+$

Reductive aminations can be achieved relatively easily without enzymes, but relatively high ammonia concentrations are necessary to allow imine reduction to the amino acid to outdo ketone reduction to the hydroxy acid. Thus, in work purporting to show that amino acids could be synthesised in deep-sea vents, a grossly unrealistic concentration of ammonia (0.375 M) was employed [43] (the concentration of ammonia in the early ocean was probably more than 5000 x lower [S5]). High concentrations of ammonia would play havoc with many of the other transformations required in this scheme. Thus, activated carboxylates would be ammonolysed to amides preventing eg. reaction 13 and 14 and thus purine ribonucleotide synthesis and converting 1,3-diphosphoglycerate to 3-phosphoglyceramide and thus preventing partial gluconeogenesis.

29. Carbamoyl phosphate synthetase. The enzyme uses ammonia from glutamate hydrolysis and two ATPs to convert bicarbonate to carbamyl phosphate. Using exogenous ammonia gives a slightly simpler process:

Bic + ATP = BicP + ADP

$BicP + NH_3 = Car + P_i$
$Car + ATP = CarP + ADP$

This enzyme catalyses 'one of the most remarkable reactions ever described in biological chemistry' [38]. The intermediates carboxyphosphate and carbamate are both extremely hydrolytically labile and carbamoyl phosphate can react with excess ammonia to generate urea. Unimaginable without an enzyme and no carbamoyl phosphate means no *de novo* synthesis of pyrimidine ribonucleotides and no arginine.

30. Aspartate carbamoyltransferase. Catalyses the carbamoylation of aspartate by carbamoyl phosphate:

$Asp + CarP = CarAsp + P_i$

Non-enzymatically, carbamoyl phosphate equilbrates with cyanate before both are hydrolysed, a reaction that is catalysed by carbonate [S6]. Cyanate will carbamylate amines, but how can the selective carbamoylation of aspartate possibly be achieved in the presence of other amino acids and ammonia?

31. Dihydroorotase. A binuclear metal center polarises carbonyl groups and a general acid (/base) of the enzyme mediates difficult (de)protonation as carbamyl aspartate is equilibrated with dihydroorotate and water:

$CarAsp = DHOro + H_2O$

32. Dihydroorotate dehydrogenase. A general base deprotonates C5 of dihydroorotate as hydride is transferred from C6 to an oxidised flavin generating orotate and reduced flavin:

$DHOro + FAD = Oro + FADH_2$

A really challenging reaction without enzymes in a highly reducing environment.

33. Orotate phosphoribosyltransferase. The enzyme catalyses the stereospecific magnesium ion-dependent phosphoribosylation of orotate by dissociative nucleophilic substitution via a transition state with considerable oxocarbonium ion character [S7]. This transition state is stabilised electrostatically by precisely positioned carboxylates and water is excluded from the active site thus preventing competing hydrolysis:

$Oro + PRPP = OMP + PP_i$

The background reaction is undetectable although non-enzymatic hydrolysis of PRPP is observed.

34. Orotidine monophosphate decarboxylase. An extraordinary decarboxylation giving uridine monophosphate:

$OMP = UMP + CO_2$

Unassisted by cofactors, this enzyme produces a huge rate enhancement (~10^{17}-fold) over the uncatalysed reaction which has a half-life of 78 million years [28]. The transformation can be accomplished photochemically with 254 nm irradiation [S8] although this seems unlikely at a deep-sea vent.

35. CTP synthetase. Phosphorylation of O4 of UTP followed by aminolysis by attack of the amido nitrogen of glutamine and carboxamide hydrolysis, or less favourably by free ammonia - the latter is less complex:

UTP + ATP = PUTP + ADP
PUTP + NH_3 = CTP + P_i

Another really challenging reaction in the absence of enzymes. Non-enzymatic phosphorylation of nucleotides is known, but occurs on phosphate or the 2',3'-diol both of which are more nucleophilic than O4. The conditions for these phosphorylation reactions – heating in formamide or molten urea – are completely incompatible with deep-sea vents and life.

36. Transaminase or amino acid dehydrogenase.

Pyruvate + NH_3 + NAD(P)H + H^+ = Ala + $NAD(P)^+$

See 28.

37. Citrate synthase. Claisen condensation of acetyl-CoA with oxaloacetate to form citryl-CoA followed by hydrolysis of the latter to citrate:

Acetyl-CoA + OAA = citryl-CoA
citryl-CoA + H_2O = citrate + CoASH

A suitably positioned general base generates the enolate of acetyl-CoA which the enzyme stabilises electrostatically thus favouring its formation. In water in the absence of an enzyme, a general base would simply catalyse the hydrolysis of acetyl-CoA. Even if conditions for the Claisen condensation in water could be found, selective hydrolysis of citryl-CoA in the presence of acetyl-CoA would be a daunting challenge. Oxaloacetate can be directly converted to citrate by UV irradiation [S9], but this is unlikely at a deep-sea vent.

38. Aconitase. Dehydration to cis-aconitate followed by rehydration to isocitrate:

Citrate = cis-aconitate + H_2O
cis-aconitate + H_2O = isocitrate

Coordinated general acid-base catalysis to dehydrate citrate coordinated by its hydroxyl group to an iron-sulfur cluster followed by flipping of cis-aconitate and rehydration. In the absence of an enzyme this would be a challenging transformation, particularly in the presence of better nucleophiles than water.

39. Isocitrate lyase. Retro Claisen condensation of isocitrate to give succinate and glyoxylate:

Isocitrate = succinate + glyoxylate

Another extraordinary piece of enzymology best appreciated by considering the reverse reaction in which the enzyme deprotonates a methylene group of succinate with a pK_a of about 25. The enzyme stabilises the aci form of the succinate carbanion by at least fifteen orders of magnitude [S10]! Doing this in water would not be possible even with a strong base which would anyway cause myriad destructive reactions of activated intermediates in the overall scheme.

40. Transaminase or amino acid dehydrogenase.

Glyoxylate + NH_3 + NAD(P)H + H^+ = Gly + $NAD(P)^+$

See 28.

41. Serine transhydroxymethylase. Pyridoxal phosphate dependent reaction of a stabilised glycine carbanion with formaldehyde derived from active site hydrolysis of methylene-tetrahydrofolate or, more simply free formaldehyde:

Gly + CH_2O = Ser

A difficult reaction without pyridoxal phosphate.

42. Isocitrate dehydrogenase. Oxidation of isocitrate to oxalosuccinate followed by decarboxylation:

Isocitrate + $NADP^+$ = oxalosuccinate + NADPH + H^+
oxalosuccinate = 2-KG + CO_2

The enzyme uses a magnesium ion to facilitate both the oxidation and the decarboxylation. The decarboxylation would occur spontaneously in the absence of an enzyme if a means of oxidising the alcohol selectively in a reducing environment could be found. The spontaneous decarboxylation (like that of OAA) would be catalysed by divalent metal ions such as magnesium ions.

43. Transaminase or amino acid dehydrogenase.

2-KG + NH_3 + NAD(P)H = Glu + $NAD(P)^+$

See 28.

44. Glutamyl-5-phosphate synthetase. Selective phosphorylation of the 5-carboxylate of glutamate:

Glu + ATP = GluP + ADP

This particular acyl phosphate is more labile than most and undergoes extremely facile cyclisation to pyroglutamate. To avoid this, it is passed to the next enzyme in the sequence in a process that avoids free diffusion, probably via an enzyme-substrate-enzyme complex. In the absence of enzyme, any reagent capable of phosphorylating carboxylate groups would do so indiscriminately.

45. Glutamyl-5-phosphate reductase. Reduction of glutamyl-5-phosphate to glutamate-5-semialdehyde which spontaneously equilibrates with Δ^1-pyrroline-5-carboxylate:

GluP + NADPH + H^+ = GluSa + P_i + $NADP^+$
GluSa = P5C + H_2O

A difficult reduction in the absence of enzymes given the propensity of glutamyl-5-phosphate to cyclise to pyroglutamate.

46. Δ^1-Pyrroline-5-carboxylate reductase. Nicotinamide mediated reduction of the iminium ion of the protonated pyrroline:

Δ^1-P5C + NAD(P)H + H^+ = Pro + $NAD(P)^+$

47. N-Acetylglutamate synthetase. Selective acetylation of the amino group of glutamate by acetyl-CoA:

Acetyl-CoA + Glu = AcGlu + CoASH

In the absence of enzymes, any form of activated acetate capable of effecting this transformation would also acetylate the amino group of other amino acids as well as other amines and ammonia.

48. N-Acetyl-glutamyl-5-phosphate synthetase. Selective phosphorylation of the 5-carboxylate of N-acetylglutamate:

AcGlu + ATP = AcGluP + ADP

Another hydrolytically labile acyl phosphate.

49. N-Acetyl-glutamyl-5-phosphate reductase. Reduction of N-acetyl-glutamyl-5-phosphate to the corresponding semialdehyde:

AcGlu + NADPH + H^+ = AcGluSa + P_i + $NADP^+$

Again, a difficult reduction non-enzymatically in part due to competing hydrolysis of the acyl phosphate.

50. N^2-Acetylornithine aminotransferase. Pyridoxal phosphate dependent transamination using glutamate as amino group donor:

AcGluSa + Glu = AcOrn + 2-KG

Without cofactor, a difficult transformation to achieve non-enzymatically, particularly with any substrate selectivity.

51. N^2-Acetylornithinase. Hydrolytic deacetylation by a divalent cation-dependent mechanism:

AcOrn + H_2O = Orn + AcO^- + H^+

Deacetylation can be relatively easily catalysed without an enzyme, but deacetylating N^2-acetylornithine selectively without cleaving other amide bonds and more labile compounds such as thioesters and acyl phosphates is really hard to imagine. Of the other amides, the formyl amide intermediates of purine assembly would be particularly prone to competing deacylation.

52. Ornithine carbamoyltransferase. Highly substrate-selective carbamoylation using carbamoyl phosphate:

Orn + CarP = CarOrn + P_i

Under conditions in which the amino group of aspartate is carbamylated (reaction 30), how could the 5-amino group of ornithine possibly be carbamoylated selectively over the 2-amino group and how could this be achieved in the presence of other amino acids and ammonia?

53. Argininosuccinate synthetase. Adenylation of the ureido oxygen of citrulline followed by displacement of inorganic phosphate by the amino group of aspartate:

CarOrn + ATP = AdCarOrn + PP_i
AdCarOrn + Asp = ArgS + AMP

Adenylating or phosphorylating the ureido oxygen of citrulline in water in the absence of an enzyme is unbelievably challenging. Urea can be O-phosphorylated transiently in dry-state melts, but such conditions are not compatible with the other transformations of the scheme, or indeed with deep-sea vents or life.

54. Argininosuccinate lyase. Elimination of a poor leaving group from argininosuccinate by general acid-base catalysis by two precisely positioned enzyme residues:

ArgS = Arg + fumarate

Another elimination reaction that is likely to be extremely slow in the absence of an enzyme.

55. Acetolactate synthase. Thiamine pyrophosphate mediated addition of an acetyl anion equivalent derived from one pyruvate to a second pyruvate in a reaction that also requires a divalent metal ion:

Pyruvate + TPP + H^+ = He-TPP + CO_2
He-TPP + pyruvate = AcLac + TPP

A chemically daunting transformation in the absence of an umpolung cofactor. In the absence of enzymes, divalent metal ions catalyse the unwanted aldol dimerisation of pyruvate [33].

56. Ketol-acid reductoisomerase. A hydroxide ion bridging two magnesium ions deprotonates the hydroxyl group of acetolactate and promotes a simultaneously general acid-catalysed [1,2]-sigmatropic shift of a methyl group to the ketone. The 2-ketoacid thus formed is then reduced by a nicotinamide cofactor to 2,3-dihydroxyisovalerate:

AcLac = 3-hydroxy-2-ketoisovalerate
3-hydroxy-2-ketoisovalerate + NADPH + H^+ = 2,3-DHiV + $NADP^+$

The initial isomerisation equilibrium strongly favours acetolactate so the enzyme uses precise positioning to target the reduction of the carbonyl group of 3-hydroxy-2-ketoisovalerate. In the absence of the enzyme, acetolactate is prone to decarboxylation, a reaction catalysed by divalent metal ions.

57. Dihydroxyacid dehydratase. Iron-sulfur cluster and general base-catalysed dehydration of 2,3-dihydroxyisovalerate to the enol of 2-ketoisovalerate followed by keto-enol tautomerisation:

2,3-DHiV = 2-KiV enol form + H_2O
2-KiV enol form = 2-KiV keto form

Like the elimination of water catalysed by aconitase, a demanding elimination of a poor leaving group.

58. Transaminase or amino acid dehydrogenase.

2-KiV + NH_3 + NAD(P)H + H^+ = Val + $NAD(P)^+$

See 28.

59. 2-Isopropylmalate synthase. With a mechanism similar to citrate synthase, this enzyme catalyses Claisen condensation of acetyl-CoA with 2-ketoisovalerate to form 2-isopropylmalyl-CoA followed by hydrolysis of the latter to 2-isopropylmalate:

Acetyl-CoA + 2KiV = 2-iPM-CoA
2-iPM-CoA + H_2O = 2-iPM + CoASH

See 37.

60. Isopropylmalate dehydratase. With a mechanism similar to aconitase, this enzyme catalyses the (de)hydrative equilibration of 2- and 3-isopropylmalate via 2-isopropylmaleate:

2-iPM = 2-isopropylmaleate + H_2O
2-isopropylmaleate + H_2O = 3-iPM

See 38.

61. 3-Isopropylmalate dehydrogenase. With a mechanism similar to isocitrate dehydrogenase, this enzyme oxidises 3-isopropylmalate to 2-keto-3-carboxyisocaproate followed by decarboxylation to 2-ketoisocaproate:

3-iPM + NAD^+ = 2-keto-3-carboxyisocaproate + NADH + H^+
2-keto-3-carboxyisocaproate = 2-KiC + CO_2

See 42.

62. Transaminase or amino acid dehydrogenase.

2-KiC + NH_3 + NAD(P)H + H^+ = Leu + $NAD(P)^+$

See 28.

63. Aspartyl-4-phosphate synthetase. Selective phosphorylation of the 4-carboxylate of aspartate:

Asp + ATP = AspP + ADP

Another hydrolytically labile acyl phosphate.

64. Aspartyl-4-phosphate reductase. Reduction of aspartyl-4-phosphate to aspartate-4-semialdehyde:

AspP + NAD(P)H + H^+ = AspSa + P_i + $NAD(P)^+$

A difficult reduction in the absence of enzymes given the hydrolytic lability of aspartyl-4-phosphate. In a system in which N-acetylglutamate-5-semialdehyde is reductively aminated to N^2-acetylornithine, it would be difficult to prevent the reductive amination of aspartate semialdehyde.

65. Homoserine dehydrogenase. Nicotinamide mediated reduction of aspartate semialdehyde to homoserine:

AspSa + NAD(P)H + H^+ = hSer + $NAD(P)^+$

In a system in which this reduction is somehow effected non-enzymatically, it would be difficult to avoid the corresponding reduction of other aldehydes, for example N-acetylglutamate-5-semialdehyde.

66. Homoserine kinase. Substrate-selective phosphorylation of homoserine to homoserine phosphate:

hSer + ATP = hSerP + ADP

A difficult enough phosphorylation to achieve in water anyway, but without a substrate-selective catalyst, how could it be achieved selectively in the presence of other primary alcohols such as glycerol phosphate?

67. Threonine synthase. Molecular gymnastics initiated by this enzyme using pyridoxal phosphate to eliminate phosphate from homoserine phosphate giving an intermediate which is hydrated at the C3 to give, after separation from the cofactor, threonine:

hSerP + H_2O = Thr + P_i

Even if the elimination of phosphate could somehow be catalysed without the enzyme and cofactor (and it looks difficult), hydration of the resultant vinylglycine would be a formidable challenge.

68. Glycerol-3-phosphate dehydrogenase. Reduction of dihydroxyacetone phosphate by a reduced nicotinamide cofactor:

DHAP + NADH + H^+ = glycerol-P + NAD^+

How could non-enzymatic reduction of a ketone be effected selectively in the presence of aldehydes such as N-acetylglutamate-5-semialdehyde?

69. Acetyl-CoA carboxylase. This enzyme uses ATP to couple bicarbonate and biotin-carboxyl carrier protein to give N^1-carboxybiotin-CCP which then carboxylates the enolate of acetyl-CoA to give malonyl-CoA:

Biotin-CCP + HCO_3^- + ATP = ^-O_2C-biotin-CCP + ADP + P_i
Acetyl-CoA + ^-O_2C-biotin-CCP = Mal-CoA + biotin-CCP

Another example of coupling ATP hydrolysis to a thermodynamically very unfavourable reaction to drive it in the synthetic direction.

70-74. Fatty acid synthetase. A molecular factory line that catalyses the stepwise assembly of fatty acids two carbons at a time. The acyl-CoA building blocks for this assembly are first attached to an acyl carrier protein (ACP) by thioester exchange which enables intermediates to be passed around the various active sites that catalyse the individual steps.

70. 3-Ketoacyl-ACP synthetase. Decarboxylation of malony-ACP generates an enolate which reacts with the acetyl thioester in a Claisen reaction giving acetoacetyl-ACP:

Ac-ACP + Mal-ACP = AcAc-ACP + CO_2

71. 3-Ketoacyl-ACP reductase. Selective reduction of the 3-keto group of acetoacetyl-ACP by nicotinamide giving 3-hydroxybutyryl-ACP:

AcAc-ACP + NADPH + H^+ = 3-HBut-ACP + $NADP^+$

72. 3-Hydroxyacyl-ACP dehydratase. Dehydration of 3-hydroxybutyryl-ACP to give crotonyl-ACP:

3-HBut-ACP = Crot-ACP + H_2O

73. Enoyl-ACP reductase. Selective reduction of crotonyl-ACP by nicotinamide to give butyryl-ACP:

Crot-ACP + NADPH + H^+ = But-ACP + $NADP^+$

74. Thioesterase. Hydrolytic cleavage of the fatty acyl-ACP generated by elongating iterative cycles of reactions 70-73:

FA-ACP + H_2O = FA + ACP

Biology confounds the arithmetic demon associated with multistep linear synthesis by catalysing each step of this process in extremely high yield. Achieving this chemically is highly challenging – rather than Claisen reactions, thioesters exposed to base in water are simply expected to hydrolyse – but even if it could be realised with each step proceeding in 50% yield, the overall yield of, for example decanoic acid, would be < 0.001%.

75. Acetyl-CoA synthase. In an enzymatic variant of the Monsanto acetic acid process, a methyl group from a cobalt corrinoid iron-sulfur protein (CoFeSP) is coupled at a nickel sulfide centre with carbon monoxide giving an acyl nickel species which is thiolysed by the thiol of CoASH:

Me-Co(III)FeSP + CO + CoASH = Acetyl-CoA + Co(I)FeSP

Non-enzymatically, in the presence of freshly precipitated iron and nickel sulfides, methane thiol and carbon monoxide react to give acetate via an activated form thereof as demonstrated by trapping experiments with aniline [42]. However, it has been demonstrated that methane thiol found in seafloor hydrothermal fluids, and originally thought to be of geochemical origin, is actually a product of biological processes [S11]. Prebiotically plausible non-enzymatic (activated) acetate synthesis using methyl donors other than methane thiol has not been reported.

76. Carbon dioxide reduction.

In an enzymatic version of the water-gas shift reaction, hydrogenase equilibrates hydrogen with protons and electrons which are transferred to carbon monoxide dehydrogenase which catalyses the reduction of carbon dioxide to carbon monoxide:

$H_2 = 2H^+ + 2e^-$
$CO_2 + 2H^+ + 2e^- = CO + H_2O$

Alternatively, and subject to thermodynamic limitation, carbon dioxide can be reduced to formate by a dehydrogenase:

$CO_2 + NADH = HCO_2^- + NAD^+$

Difficult transformations to achieve non-enzymatically with any degree of efficiency under conditions compatible with the other reactions of the overall scheme for kinetic and thermodynamic reasons. The presence of iron-sulfur clusters in these enzymes has been interpreted as a sign of their antiquity. Speculation also has it that this implies a role for iron-sulfur minerals in catalysing non-enzymatic underlying chemistry at the origin of life. Since these clusters also bear cyanide ligands, the same line of reasoning would suggest the presence of cyanide at such a time. As cyanide is not a likely component of deep-sea vent hydrothermal fluid, this would suggest that, if such reasoning is correct, the underlying chemistry took place at a surface location. Using simulated vent conditions with precipitates of iron and nickel sulfides, the reduction of carbon dioxide to low micromolar concentrations of formate and low nanomolar concentrations of formaldehyde has been claimed. Further claims that the degree of reduction was influenced by pH gradients in the system have been refuted [S12].

Reduction of formaldehyde to methanol is plausible and mimics alcohol dehydrogeanse chemistry, but conversion of methanol to methane thiol under prebiotic conditions is highly demanding.

77. Nitrogen feedstock reduction.

Nitrogenase. The enzymes that collectively convert molecular nitrogen to ammonia do more than simply bring about the reduction as the process is coupled to ATP hydrolysis and also produces hydrogen [S13]:

$N_2 + 8e^- + 8H^+ + 16ATP = 2NH_3 + H_2 + 16\ ADP + 16P_i$

The simple reduction of nitrogen by hydrogen can be achieved by the Bosch-Haber process, but the conditions are not prebiotically plausible or compatible with the transformations of the overall scheme.

More plausibly, nitrite and nitrate produced in water under an atmosphere containing nitric oxide, due to lightning or impact shock, can be reduced to ammonia by ferrous ions. It is estimated that the concentration of ammonia in sea water would have been $< 70\ \mu M$ [S5],

more than five thousand times lower than the 0.375 M required to synthesise alanine by reductive amination under conditions otherwise simulating a deep-sea vent [43].

Supplementary references

S1 Weber, J.P. and Bernhard, S.A. (1982) Transfer of 1,3-diphosphoglycerate between glyceraldehyde-3-phosphate dehydrogenase and 3-phosphogycerate kinase via an enzyme-substrate-enzyme complex. *Biochemistry* **21**, 4189–4194.

S2 Hove-Jensen, B., Andersen, K.R., Kilstrup, M., Martinussen, J., Switzer, R.L. and Willemoës, M. (2017) Phosphoribosyl diphosphate (PRPP): Biosynthesis, enzymology, utilization and metabolic significance. *Microbiol. Mol. Biol. Rev.* **81**, 1–83.

S3 Rudolph, J. and Stubbe, J. (1995) Investigation of the mechanism of phosphoribosylamine transfer from glutamine phosphoribosylpyrophosphate amidotransferase to glycinamide ribonucleotide synthetase. *Biochemistry* **34**, 2241–2250.

S4 Meyer, E., Leonard, N.J., Bhat, B., Stubbe, J. and Smith, J.M. (1992) Purification and characterisation of the *pur*E, *pur*K and *pur*C gene products: Identification of a previously unrecognized energy requirement in the purine biosynthetic pathway. *Biochemistry* **31**, 5022–5032.

S5 Summer, D.P. and Chang, S. (1993) Prebiotic ammonia from reduction of nitrite by iron (II) on the early Earth. *Nature* **365**, 630–633.

S6 Vogels, G.D., Uffink, L. and van der Drift, C. (1970) Cyanate decomposition catalyzed by certain bivalent anions. *Recueil* **89**, 500–508.

S7 Tao, W., Grubmeyer, C. and Blanchard, J.S. (1996) Transition state structure of *Salmonella typhimurium* orotate phosphoribosyltransferase. *Biochemistry* **35**, 14–21.

S8 Ferris, J.P. and Joshi, P.C. (1979) Chemical evolution 33. Photochemical decarboxylation of orotic acid, orotidine and orotidine-5'-phosphate. *J. Org. Chem.* **44**, 2133–2137.

S9 Waddell, T.G., Geevarghese, S.K., Henderson, B.S., Pagni, R.M. and Newton, J.S. (1989) Chemical evolution of the citric acid cycle: Sunlight and ultraviolet photolysis of cycle intermediates. *Orig. Life Evol. Biosph.* **19**, 603–607.

S10 Schloss, J.V. and Cleland, W.W. (1982) Inhibition of isocitrate lyase by 3-nitropropionate, a reaction-intermediate analogue. *Biochemistry* **21**, 4420–4427.

S11 Reeves, E.P., McDermott, J.M. and Seewald, J.S. (2014) The origin of methanethiol in midocean ridge hydrothermal fluids. *Proc. Natl. Acad. Sci. USA* **111**, 5474–5479.

S12 Jackson, J.B. (2017) The "origin-of-life reactor" and reduction of CO_2 by H_2 in inorganic precipitates. *J. Mol. Evol.* **85**, 1–7.

S13 Hoffmann, B.M., Lukoyanov, D., Yang, Z.-Y., Dean, D.R. and Seefeldt, L.C. (2014) Mechanism of nitrogen fixation by nitrogenase: The next stage. *Chem. Rev.* **114**, 4041–4062.
